# Supplementary material for: Electronic reminders and rewards to improve adherence to inhaled asthma treatment in adolescents: a non-randomised feasibility study in tertiary care
Source: BMJ Open. 2021 Oct 28;11(10):e053268. doi: 10.1136/bmjopen-2021-053268 (PMC8559117; doi:10.1136/bmjopen-2021-053268)
Supplement: Supplementary data [file bmjopen-2021-053268supp001.pdf]

SUPPLEMENTARY MATERIAL

Participant	N.1				N.2				N.3				N.4				N.5				N.6				N.7				N.8				N.9				N.10			
**Visit**	**1**	**2**	**3**	**4**	**1**	**2**	**3**	**4**	**1**	**2**	**3**	**4**	**1**	**2**	**3**	**4**	**1**	**2**	**3**	**4**	**1**	**2**	**3**	**4**	**1**	**2**	**3**	**4**	**1**	**2**	**3**	**4**	**1**	**2**	**3**	**4**	**1**	**2**	**3**	**4**
How much affects your life	7	6	7	5	3		3	3	4		2		6	5			6				1	1			7	3	2		2	7	3		8	7	7		2		2	
How long your illness will continue	5	7	5	6	10		7	10	5		4		8	10			9				6	6			3	3	3		3	4	3		7	8	5		5		5	
How much control do you have	5	5	5	7	10		10	9	4		6		5	9			8				9	8			8	7	7		3	9	8		3	5	3		7		7	
How much treatment can help	6	8	6	7	9		9	10	5		6		7	9			10				9	10			10	8	9		6	10	8		ni	1	2		8		9	
How much symptoms you experience	6	6	5	5	3		4	6	4		5		5	5			7				4	1			5	1	1		5	7	6		9	8	8		3		3	
How concern you are	7	7	7	5	1		1	4	5		3		10	10			8				5	3			3	0	1		5	10	7		4	2	2		4		4	
How well you understand your illness	7	8	8	6	7		5	5	5		10		6	10			9				10	9			10	9	10		8	9	8		10	10	10		8		8	
How much affects you emotionally	3	8	5	7	0		1	1	4		0		3	10			7				0	1			2	0	0		4	3	1		3	0	1		1		3	

Supplementary Table 1. Brief illness perception questionnaire (B-IPQ).
0= not at all, 10= extremely.

**Participant**	**N.1**				**N.2**				**N.3**				**N.4**				**N.5**				N.6				**N.7**				**N.8**				**N.9**				**N.10**			
**Visit**	**1**	**2**	**3**	**4**	**1**	**2**	**3**	**4**	**1**	**2**	**3**	**4**	**1**	**2**	**3**	**4**	**1**	**2**	**3**	**4**	**1**	**2**	**3**	**4**	**1**	**2**	**3**	**4**	**1**	**2**	**3**	**4**	**1**	**2**	**3**	**4**	**1**	**2**	**3**	**4**
1) My health depends on asthma medicines	2	3	4	3	3		3	2	3		3		3	2			2				1	3			2	4	4		1	1	1		3	5	4		2		1	
2) Having to take asthma medication worries me	3	4	4	4	5		5	5	4		4		4	1			4				5	4			4	4	4		5	5	3		4	5	4		5		4	
3) My life would be impossible without my asthma medication	1	2	3	2	4		4	4	3		2		2	1			2				2	3			3	5	4		3	2	3		2	5	5		1		2	
4) Without my asthma medication I would be very ill	2	2	3	4	4		4	3	2		2		2	1			2				3	2			5	1	3		2	1	2		1	3	4		2		2	
5) I sometimes worry about the long-term effects of my asthma medication	2	2	2	2	2		4	4	4		4		3	1			2				4	4			4	4	3		2	3	3		4	5	4		5		3	
6) My asthma medication is mystery to me	3	4	4	4	5		4	4	4		4		3	3			4				5	5			5	2	3		5	5	4		2	2	1		4		5	
7) My health in the future will depend on my asthma medication	3	3	3	3	4		5	3	4		4			1			3				2	4			ni	5	3		1	2	2		4	ni	4		3		3	
8) My asthma medication disrupts my life	4	2	4	4	5		5	4	4		4			4			4				5	5			4	4	4		5	5	4		4	ni	1		5		4	
9) I sometimes worry about becoming too dependent on my asthma medication	2	2	2	4	4		4	4	4		4			4			2				3	3			2	4	3		3	3	4		2	ni	3		5		3	
10) My asthma medication protects me from becoming worse.	2	2	2	2	2		2	2	2		2			1			2				1	1			1	3	4		1	1	1		2	ni	4		1		1	
11) Doctors use too many medicines	1	2	1	2	5		5	5	4		3			3			4				5	5			3	3	3		2	4	3		1	ni	1		4		3	
12) People who take medicines should stop their treatment for a	2	2	2	2	2		2	3	4		3			4			4				3	3			5	5	5		5	5	5		2	ni	1		3		3	

while every now and again																																								
13) Most medicines are addictive	3	5	4	4	4		5	5	2		3			3			4				4	4			4	4	4		5	5	3		3	2	3		5		3	
14) Natural remedies are safer than medicines	1	2	1	2	3		4	4	3		3			3			4				4	4			ni	3	3		5	3	5		2	3	2		2		2	
15) Medicines do more harm than good	2	2	2	2	5		5	5	4		3			4			4				5	5			4	4	3		5	5	5		3	3	3		3		3	
16) All medicines are poisons	2	2	3	2	5		5	5	4		3			4			4				5	5			5	5	5		5	5	5		4	3	3		5		4	
17) Doctors place too much trust on medicines	2	2	1	2	5		5	5	4		3			4			4				5	3			4	3	4		4	4	3		2	2	1		4		4	
18) If doctors had more time with patients, they would prescribe fewer medicines	3	2	1	2	4		5	5	4		3			4			3				5	4			2	2	3		5	5	5		3	3	3		3		3	

Supplementary Table 2. Beliefs about Medicines questionnaire (BMQ).
1= strongly agree, 2= agree, 3= uncertain, 4= disagree, 5= strongly disagree
ni = not input

BMJ Publishing Group Limited (BMJ) disclaims all liability and responsibility arising from any reliance placed on this supplemental material which has been supplied by the author(s)

**Outcome**	**Visit N.**	**Pt1**	**Pt2**	**Pt3**	**Pt4**	**Pt5**	**Pt6**	**Pt7**	**Pt8**	**Pt9**	**P10**
**Adherence (%)**	0	46	74	59	70	3	49	45	59	54	72
	1	*n/a*	*n/a*	*n/a*	*n/a*	*n/a*	*n/a*	*n/a*	*n/a*	*n/a*	*n/a*
	2	44	83	96	45		61	72	81	58	58
	3	44	77	68			83	82	92	7	58
	4	39	63	47			71	27	91	17	59
**ACT**	1	13	23	21	19	15	25	22	15	9	23
	2	17			18		25	23	25	10	
	3	18	21					21	25	10	23
	4	19	20								
**FeNO**	1	44	68	20	77		24	59	22	40	
	2	93		15	18		26	20	13	49	
	3	107	50	35				22	19	54	13
	4	56	78								
**FEV1**	1 pre	2.04	3.58	2.44	2.62	2.22	2.79	2.03	1.88	2.21	2.09
	post	2.06	3.70	3.78		2.37		2.32	2.44	2.67	2.11
	2 pre	1.92		3.86	3.08		2.75	2.63	2.04	2.29	
	post	1.92		3.86			2.78	2.45	2.32	2.61	
	3 pre	1.71	3.91	3.54				2.55	2.19		2.23
	post	2.01	4.14	3.91				2.42	2.30		2.36
	4 pre	1.92									
	post	1.80									

Supplementary Table 3. Outcomes collected during the study. Pt = patient, adherence visit 0 = measured prior to entering the study; ACT = asthma Control Score; FeNO = fractional exhaled nitric oxide; FEV1 = forced expiratory volume.

FEV1 pre and post beta-agonists. Single values indicate spirometry performed without beta-agonists.
